# Rotator cuff tendon augmentation and inter-user reproducibility assessment of a novel patch fixation technique compared to a conventional technique for rotator cuff repair: a biomechanical validation study

**DOI:** 10.1016/j.xrrt.2026.100844

**Published:** 2026-08-05

**Authors:** Bettina Hochreiter, Ronja Senn, Carmen Castroviejo, Andrea Möhl, Cherilyn Camichel, Philipp Kriechling, Peter N. Chalmers, Jonathan D. Barlow, Karl Wieser

**Affiliations:** a Department of Orthopaedics, Balgrist University Hospital, University of Zurich, Zurich, Switzerland; b ZuriMED Technologies AG, Zurich, Switzerland; c Department of Orthopaedic Surgery, University of Utah, Salt Lake City, UT, USA; d Department of Orthopedic Surgery, Mayo Clinic, Rochester, MN, USA

**Keywords:** Rotator cuff repair, Patch augmentation, Biomechanics, Reproducibility, Fiberlocker, Fiberlocking, Interlocked patch augmentation

## Abstract

**Background:**

Rotator cuff (RC) tears present a major socioeconomic burden with high rates of retear after repair surgery. The interlocked patch augmentation represents a novel approach using direct fiber interlocking of a nonwoven polyethylene terephthalate patch into tendon tissue. This study evaluated the biomechanical strength and properties of human cadaveric RC augmentation with direct patch interlocking and its reproducibility.

**Methods:**

Two complementary studies were conducted: (1) a biomechanical human cadaver study on paired infraspinatus tendons comparing 2 horizontal mattress stitches with and without interlocked patch augmentation (n = 5 per group) measuring cyclic loading and load-to-failure testing; (2) a user performance reproducibility evaluation with 6 orthopedic surgeons (first-time users group) and 3 interlocked patch augmentation experts (control group), each performing 4 arthroscopic procedures using ovine infraspinatus tendons.

**Results:**

In the human cadaver study, direct patch interlocking demonstrated significantly higher ultimate tensile strength and linear stiffness compared with the nonaugmented group (519.4 ± 150.8 N vs. 294.6 ± 84.1 N; *P* = .0195 and 44.7 ± 13.2 N/mm vs. 21.2 ± 6.4 N/mm; *P* = .0016). Cyclic stiffness and elongation did not show significant differences. In the user performance study, no significant difference in ultimate tensile strength was found between first-time users (392.1 ± 62.1 N) and experts (322.6 ± 86.3 N, *P* = .0991). Inter-user comparison among surgeons showed no significant differences (*P* = .4830).

**Conclusion:**

The interlocked patch augmentation significantly improves biomechanical suture retention properties in a human cadaveric RC model and enables reproducible outcomes when used by orthopedic surgeons without prior device training. These preliminary biomechanical findings support further evaluation of direct patch interlocking for RC repair augmentation in further biomechanical and clinical studies.

Rotator cuff (RC) tears are among the most prevalent shoulder pathologies, affecting millions of patients worldwide and representing a significant socioeconomic burden.^4^^,^^7^ Despite advances in surgical techniques and materials, retear rates following RC repair remain high, ranging from 17% to 69% depending on tear size and complexity and reported to be as high as 90%.^5^, ^6^, ^7^^,^^9^^,^^12^ The primary cause of repair failure is mechanical weakness at the suture-tendon interface, where conventional sutures often cut through compromised tendon tissue.^2^^,^^16^

Patch augmentation has emerged as a promising strategy to address these limitations by providing mechanical reinforcement and biological scaffolding to enhance repair integrity.^10^^,^^15^ However, current patch augmentation techniques using conventional suturing methods present several challenges including technical complexity, prolonged surgical time, and inconsistent medial (patch to tendon) fixation quality.^1^^,^^14^^,^^19^ These factors contribute to a steep learning curve and variable clinical outcomes. The interlocked patch augmentation is a novel approach to RC patch augmentation, utilizing a “surgical felting” technique that directly interlocks nonwoven polyethylene terephthalate (PET) patch fibers into the underlying tendon tissue.^8^^,^^13^ This approach eliminates the need for trans-tendon sutures to fix the patch on top of the underlying repair, instead creating a mechanically engaged interface through fiber interlocking across the entire patch-tendon contact area.

Previous ovine studies by our group have demonstrated the biomechanical superiority of direct patch interlocking, showing significant improvements in ultimate tensile strength and cyclic stiffness compared to both nonaugmented repairs and conventional patch techniques.^8^^,^^13^ The direct fiber interlocking mechanism resulted in 179% higher ultimate tensile strength compared to nonaugmented repairs and 165% higher strength compared to conventional patch augmentation.^8^ The same ovine studies also reported good biocompatibility, with minimal fibrosis and tissue ingrowth into the patch.^13^

Despite these promising biomechanical and biological outcomes, 2 critical aspects remain to be thoroughly evaluated for clinical translation: biomechanical efficacy in human cadaveric tendons and reproducibility of outcomes across different users. The ability to perform patch augmentation effectively and consistently is essential for widespread clinical adoption, particularly given the cost pressures and efficiency demands in modern surgical practice.

Therefore, the purpose of this study was to (1) directly compare biomechanical outcomes in RC tendon repair with and without interlocked patch augmentation in a clinically relevant human cadaver model; and (2) evaluate the reproducibility in reinforcement strength of interlocked patch augmentation outcomes when used by orthopedic surgeons without device-specific training compared to expert users.

## Materials and methods

Two separate experiments were performed using different specimens (Part 1: human cadaveric vs. Part 2: ovine), testing machines, and loading protocols. They address distinct questions and their absolute biomechanical values are not directly comparable; methods and results are therefore reported and interpreted separately.

### Part 1: biomechanical outcomes in cadaveric infraspinatus tendon repair with and without interlocked patch augmentation

#### Specimens and surgical procedures

Ten fresh-frozen human cadaver shoulders from 5 donors (mean age 60.6 ± 3.5 years; 1 male, 4 female) were obtained from Science Care (Phoenix, AZ, USA). The 2 shoulders of each donor were used as matched pairs, with one randomly assigned to the experimental group (interlocked patch augmentation, n = 5) and the contralateral shoulder to the control group (no augmentation, n = 5). Shoulders were dissected to remove all tissue except for the RC tendons and humeri. The tendons were then inspected and sharply detached from their footprint. Each tendon was inspected macroscopically for gross abnormalities; no validated histologic or imaging-based quality grading was performed. Their tendon thickness and width were measured with a caliper.

#### Control group

**Figure 1:**
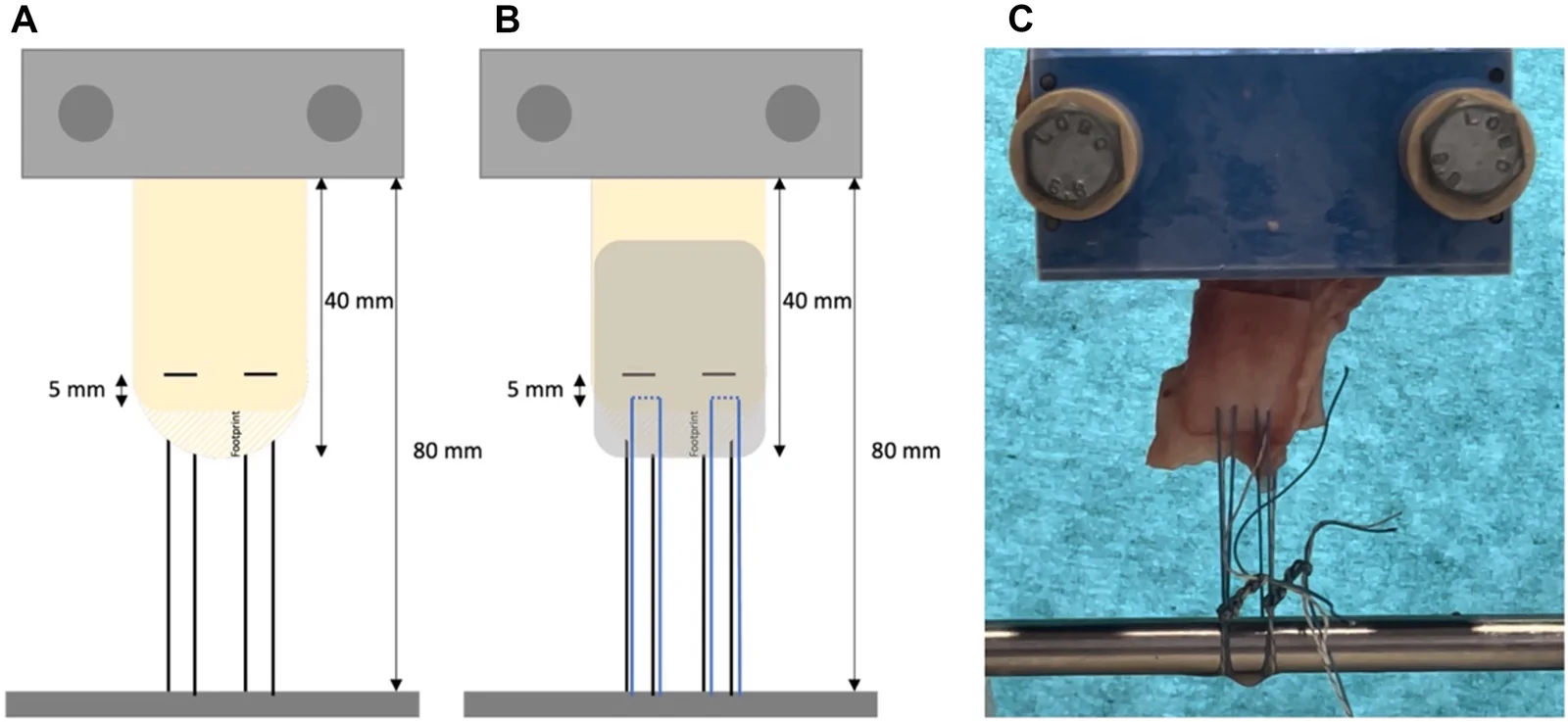
Shows the biomechanical testing setup. The upper end of the tendon was clamped into the machine setup using a custom-made cryoclamp, and the suture ends were fixed via a surgeon's knot followed by 5 alternating half-hitches around a cylindrical bolt. (**A**) Nonaugmented control group, (**B**) and (**C**) interlocked patch augmented infraspinatus tendon.

In the control group, 2 horizontal mattress stitches were placed through each infraspinatus (ISP) tendon using No. 2 FiberWire (Arthrex, Naples, FL, USA). The stitches were placed 5 mm proximal to the proximal margin of the tendon footprint, and the stitch width was set to approximately 4 mm. This approach ensured that the horizontal mattress stitches were positioned medial to the rotator cable in all tendons (Fig. 1). In cases where the tendon footprint was frayed, the damaged tissue was carefully débrided and removed to ensure a consistent amount of tendon length distal to the stitches across specimens.

#### Interlocked patch group

For the interlocked patch group, stitches were placed identically to the control group. A 25 × 30 mm nonwoven PET patch (SpeedPatch, ZuriMED Technologies AG, Zurich, Switzerland) was equipped with 2 horizontal mattress sutures (No.2 FiberWire) at its anterolateral and posterolateral edges with suture limbs exiting at the top face of the implant. The implant was soaked in phosphate-buffered saline before being positioned over the ISP tendon to cover the mattress sutures. The construct was then augmented using the FiberLocker System (ZuriMED Technologies AG, Switzerland) (Fig. 1). To replicate anatomical constraints of an in vivo setting, patch attachment was performed with the underlying cadaveric humeral head. The FiberLocker Instrument was mounted onto its compatible shaver handpiece (DYONICS POWERMAX, Smith & Nephew, London, UK), and the power unit (DYONICS POWER II) was set to 2,500 rotations per minute. The FiberLocker Instrument was applied for approximately 90 seconds at a 90° angle across the entire patch surface to create fiber interlocking.

The direct comparison in this experiment (suture-only vs. suture + interlocked patch, with an identical underlying repair in both groups) is designed to quantify the biomechanical effect of adding the interlocked patch construct to a standardized human cadaveric repair.

#### Biomechanical testing

Biomechanical properties of the repair constructs were assessed using a material testing machine (Zwick 1,456, ZwickRoell, Germany) with a 20 kN load cell. The upper end of the tendon was clamped into the machine setup using a custom-made cryoclamp, and the suture ends were fixed via a surgeon's knot followed by 5 alternating half-hitches around a cylindrical bolt (Fig. 1). For the interlocked patch group, all 8 suture limbs were secured around the cylindrical bolt. The grip-to-grip separation was consistently maintained at 80 mm. The tensile testing comprised a primary cyclic loading and subsequent destructive pull-to-failure testing similar to the testing protocol described by Jung et al.^10^ Following a preload of 20N for 2 minutes, the specimens were cyclically loaded from 10N to 30N for 10 cycles at a rate of 1 Hz. Subsequently, the specimens were linearly pulled to failure at 1 mm/s. The ultimate tensile strength (UTS) [N], defined as the maximum force that the specimens could sustain prior to a substantial drop, was measured. The mode of failure was noted for each specimen. All calculations were performed in Python 3.13.0 (Python Software Foundation, DE, USA).

## Statistical analysis

For statistical analysis, GraphPad Prism 9.5.1 (GraphPad Software, San Diego, CA, USA) was used. The level of statistical significance was set to *P* < .05. The mechanical outcome measures of the control and interlocked patch groups are compared. Normality is tested using the Shapiro-Wilk normality test. A paired *t*-test is used if a normal distribution of the differences between pairs is given. A Wilcoxon signed-rank test is used for non-Gaussian distribution.

### Part 2: reproducibility of biomechanical outcomes

#### Participants

The “first-time users group” consisted of 6 orthopedic surgeons with backgrounds in arthroscopic shoulder surgery (Table I). The “control group” comprised 3 members of the device research and development team with 3-6 years of experience with the interlocking technique. They were device developers rather than independent practicing shoulder surgeons, which limits their use as an unbiased expert benchmark.

**Table I:** Participant characteristics—first-time users and ultimate tensile strength results by participant.

ID	Group	Current role	Practice location	RCR/year	Years in practice	UTS mean ± SD (N)	Patch failures	Interface failures
S1	First-time user	Attending physician	Utah (US)	100–150	6–10	368.3 ± 170.4	3	1
S2	First-time user	Attending physician	Mayo (US)	51–100	6–10	322.8 ± 152.5	2	2
S3	First-time user	Fellow	Balgrist (CH)	1–20	1–5	342.5 ± 136.9	3	1
S4	First-time user	Consultant	Balgrist (CH)	51–100	6–10	384.0 ± 71.1	3	1
S5	First-time user	Chief resident	Balgrist (CH)	1–20	6–10	466.0 ± 87.7	3	1
S6	First-time user	Resident	Balgrist (CH)	1–20	1–5	469.0 ± 124.5	4	0
C1	Experienced user	R&D team		–	3–6 (device)	345.8 ± 147.2	3	1
C2	Experienced user	R&D team		–	3–6 (device)	322.0 ± 40.7	4	0
C3	Experienced user	R&D team		–	3–6 (device)	300.0 ± 51.2	1	3

*SD*, standard deviation.

#### Test setup and procedure

A validated arthroscopic shoulder simulator was used, featuring a real-size humerus (Synbone AG, Zizers) fixed at 30° elevation with 6 arthroscopic portals equipped with cannulas (PassPort Button Cannula, 10 mm ID x 4 cm, Arthrex, Naples, FL, USA). Fresh ovine ISP tendons served as the surgical model, positioned to replicate human supraspinatus anatomy.

Twenty-four fresh sheep shoulders (aged 6-8 months) were obtained from the local butcher (Metzgerei Angst, Zurich, Switzerland) and dissected fresh (on the same day) to remove all tissue except the ISP tendons and humeri. The infraspinati were cut approximately 8 cm from their bony insertion point. All muscle tissue was carefully removed from the tendons. The tendons were visually inspected for abnormalities and excluded if any were detected. The ISPs and humeri were then stored in a phosphate-buffered saline-soaked cloth at −20 °C until use. Each specimen was allowed to thaw at room temperature of 25 °C for 2 hours before instrumentation and testing. The ISP tendons were sharply detached from their footprint at the greater tuberosity using a scalpel.

**Figure 2:**
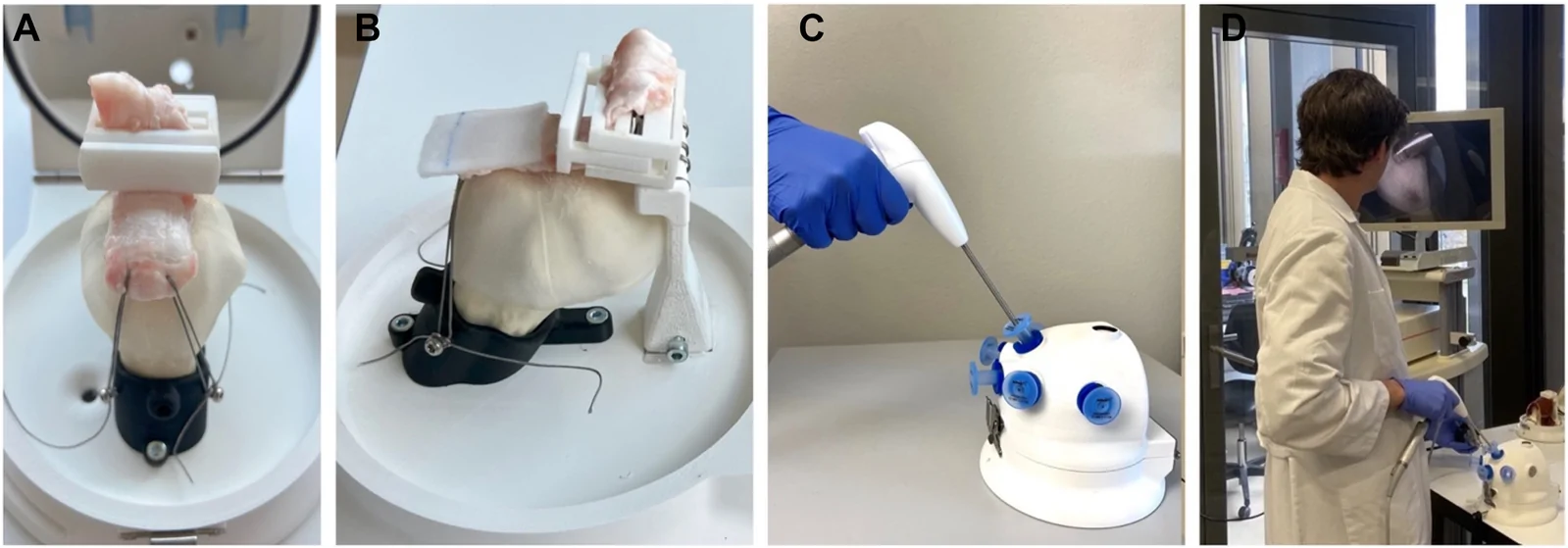
(**A**) Fixation of the ISP to the shoulder simulator using a custom clamp and simple stitches. (**B**) Placement of the interlocked patch over the ISP tendon with 0.75 cm lateral overhang for biomechanical testing. (**C**) Arthroscopic simulator and FiberLocker instrument. (**D**) Test set-up. *ISP*, infraspinatus.

For mounting the tendon to the shoulder simulator, the proximal end was secured to a custom-designed clamp (Fig. 2). Two simple stitches were passed through the lateral aspect of the tendon and secured at the humerus, covering the entire footprint of the physiological supraspinatus tendon.

The nonwoven PET patch (SpeedPatch) was placed on top of the tendon. The patch position copied its location during clinical use, where the patch is placed to cover the entire footprint and laterally overlaps the tendon insertion slightly. In this case, the lateral overhang was specified at 0.75 cm to allow for later patch clamping for the biomechanical assessment (Fig. 2). After placing the ovine tendon and the patch, the arthroscopic simulator could be closed. The arthroscopic simulator was equipped with a camera system and arthroscope (Smith and Nephew 560 HD Camera System, Smith & Nephew, London, UK) to allow for an arthroscopic setup (Fig. 2). The FiberLocker Instrument was mounted onto its compatible shaver handpiece (Dyonics Powermax, Smith & Nephew, London, UK), and the power unit (Dyonics Power II) was set to 2,500 rotations per minute.

All participants were handed the instructions for use and the “surgical technique” guide. A standardized introduction was given to each participant outlining the study procedure and their task. The only task to be performed by the participant was the fixation of the patch to the tendon achieved using the FiberLocker Instrument through the arthroscopic portal. The recommended application time was 90 seconds; however, participants relied on their surgical expertise to determine procedure completion without external guidance. Each participant performed 4 sequential procedures resulting in a total of 24 specimens in the surgeons' group and 12 specimens in the control group.

#### Biomechanical testing

A calibrated universal material testing machine Z010 (ZwickRoell GmbH, Ulm, Germany) was used for the biomechanical testing. The tendon end was wrapped in a layer of gauze and fixed in the upper tendon clamp while the lower part of the machine clamped the patch. The loading protocol consisted of a preload and subsequent destructive loading.^3^ Testing was initiated with a preload of 5 N for 60 seconds (position controlled). Following this, the sample underwent a pull-to-failure test at a speed of 1 mm/s. For each specimen, the mode of failure was noted. Interface failure describes the repair construct failing at the suture-tendon interface while the sutures have torn through the tendon but the patch remained attached to tendon. Patch failure was noted if the patch detached from its original fixation location and dislodged from the underlying tendon. The load-displacement curve was recorded by TestXpert II software (ZwickRoell GmbH, Ulm, Germany). Based on the recorded raw data, the ultimate tensile strength was computed for each specimen.

## Statistical analysis

Statistical analyses were performed using RStudio Version 4.3.1 (Posit, Boston, MA, USA) and GraphPad Prism 9.5.1. For the user-performance study, the ultimate tensile strength of the first-time user group was compared with the experienced-user group using an unpaired *t*-test, and the 6 first-time users were compared with one another using a one-way analysis of variance. Neither is an equivalence test, and no a priori power calculation or prespecified equivalence margin was defined. Statistical significance was set at *P* < .05.

## Results

### Part 1: biomechanical outcomes in cadaveric infraspinatus tendon repair with and without interlocked patch augmentation

**Figure 3:**
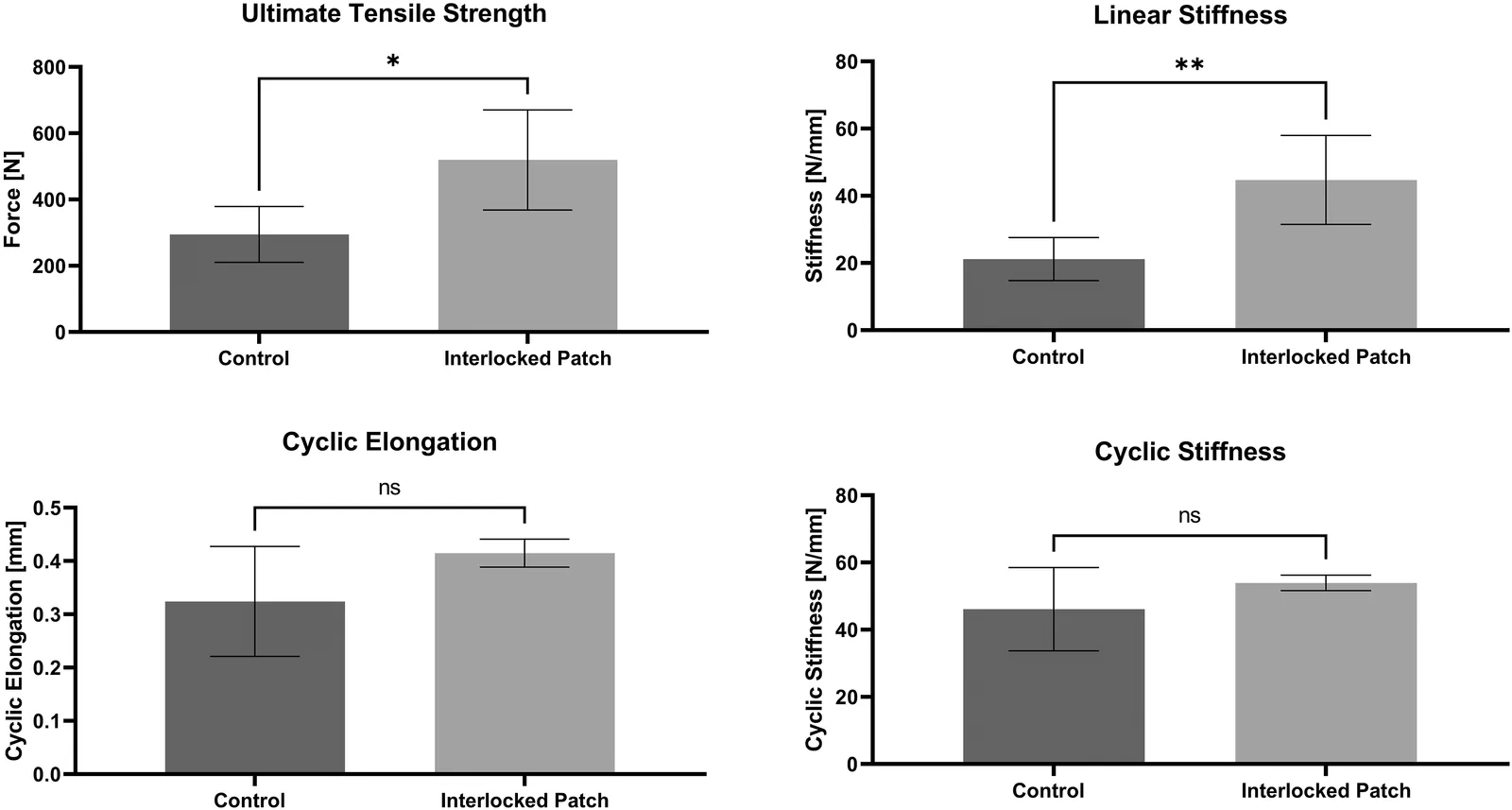
Comparison of biomechanical properties with and without interlocked patch augmentation. *ns*, not significant; ∗, <.05; ∗∗, <.005.

There was no significant difference in tendon thickness between groups (control: 6.6 mm ± 0.9; interlocked patch: 6.8 mm ± 1.8). Direct patch interlocking significantly increased UTS (519.4 ± 150.8 N vs. 294.6 ± 84.1 N; *P* = .0195) and linear stiffness (44.7 ± 13.2 N/mm vs. 21.2 ± 6.4 N/mm; *P* = .0016) compared with the nonaugmented control. Cyclic stiffness (49.2 ± 10.7 vs. 42.6 ± 13.3 N/mm; *P* = .2293) and cyclic elongation (0.5 ± 0.2 vs. 0.4 ± 0.1 mm; *P* = .2171) did not differ significantly between groups (Table II and Fig. 3). In all 5 control specimens, failure occurred by sutures cutting through the tendon. In 4 of 5 interlocked specimens, the sutures pulled through the tendon and the distal aspect of the patch while the interlocked patch-tendon interface remained fully intact; in the remaining specimen, the sutures first cut through the tendon, and the interlocked patch-tendon interface subsequently failed. Thus, the interlocked patch-tendon interface remained intact in 4 of 5 specimens, and the predominant weak point of the construct was the suture-tissue interface.

**Table II:** Comparison of biomechanical properties with and without interlocked patch augmentation.

Group	UTS (N)	Linear stiffness (N/mm)	Cyclic stiffness (N/mm)	Cyclic elongation (mm)
Control (mean ± SD)	294.6 ± 84.1	21.2 ± 6.4	42.6 ± 13.3	0.4 ± 0.1
Experimental (mean ± SD)	519.4 ± 150.8	44.7 ± 13.2	49.2 ± 10.7	0.5 ± 0.2
*P* value	.0195	.0016	.2293	.2171

*UTS*, ultimate tensile strength; *SD*, standard deviation.

### Part 2: reproducibility of biomechanical outcomes

#### Ultimate tensile strength

No significant difference in UTS was observed between first-time (392.1 ± 62.1 N) and experienced users (322.6 ± 86.3 N, *P* = .0991). Individual participant results showed consistent performance across all trials (Table I). Analysis of variance revealed no significant differences among individual participants in the first-time users group (*P* = .4830); individual participant results are shown in Table I.

## Discussion

The main findings of this study are that, in a small human cadaveric model, direct patch interlocking increased time-zero ultimate tensile strength by >170% and linear stiffness by >200% compared with a suture-only repair, and that the technique was feasible for first-time users to perform, producing fixation strengths within the range observed for experienced users.

In the cadaveric model, direct patch interlocking substantially improved the mechanical robustness of the suture–tendon construct, even in middle-aged and therefore most likely degenerative human tissue, a clinically relevant scenario where conventional repairs are most vulnerable. The marked increases in ultimate tensile strength and linear stiffness demonstrate the benefit of distributed loads across a much broader tendon through patch tendon interlocking, rather than concentrating forces at the suture strands alone. That cyclic stiffness and elongation remained comparable between groups reinforces that the augmentation primarily enhances peak-load resistance without altering early elastic behavior of the repair. Importantly, the predominance of tendon-suture cut-through as the failure mode in both groups underscores that the patch interlocking does not introduce new mechanical weak points. These findings align with prior ovine data^8^^,^^13^ demonstrating load-sharing via thousands of fiber anchoring points rather than focal suture fixation and confirm that the biomechanical benefits of patch interlocking translate to human degenerative tendon, an essential prerequisite for clinical adoption.

The user performance repeatability analysis provides evidence that first-time users, without device-specific training, achieved fixation strength that was not significantly different to expert users. The absence of significant differences in UTS between first-time users and experts indicates that the interlocked patch augmentation's design enables intuitive use based on existing surgical expertise. This finding is particularly important given the complexity often associated with structural patch fixation methods, as most of them require passing sutures through the tendon and the patch with careful attention to suture management and sequential fixation steps.^11^ The consistent performance among individual surgeons further supports the system's reproducibility. This consistency from the first trial suggests minimal learning curve requirements, contrasting with conventional patch techniques that often require extensive training and experience to achieve optimal outcomes.^14^^,^^17^^,^^19^ This is particularly important when one considers that while more than half of shoulder surgeons use patches, the majority of these surgeons have limited experience, implanting fewer than 10 patches per year.^1^ The distributed load sharing across the entire patch-tendon interface through the creation of thousands of anchoring points may contribute to more forgiving performance characteristics, allowing surgeons to achieve consistent results despite variations in technique.

The combination of mechanical augmentation, reproducible outcomes, and reduced surgical time (90-second patch fixation) suggests direct patch interlocking could offer meaningful advantages for RC repair augmentation. The elimination of device-specific training requirements and steep learning curves may lead to more reliable surgical outcomes for patients independent of the surgeons' experience. The time savings could allow surgeons to perform additional procedures within existing operating room schedules or allocate more time to other surgical steps. This efficiency gain may be particularly valuable given the projected increase in RC surgeries as the population ages.^20^ Because no procedure-timing data were collected in this study, future studies will need to be conducted to confirm that these time savings persist in the operating room.

Several limitations should be considered when interpreting these results. The user performance study utilized an ovine tendon, which, while well-established for RC research, may not fully replicate the challenges of human arthroscopic surgery. The controlled laboratory environment also differs from the complex clinical scenarios surgeons encounter in practice. In addition, the expert comparison group consisted of members of the device's research and development team rather than independent practicing surgeons; their familiarity with the technique and their involvement in its development mean they should not be regarded as a fully unbiased expert benchmark, and the inter-user comparison should be interpreted with this in mind. The observed variability is comparable to that reported in prior biomechanical studies of patch-augmented constructs and reflects inherent specimen and material heterogeneity rather than surgeon-dependent factors. In addition, the reproducibility arm used no equivalence or noninferiority design and no a priori power analysis, so the nonsignificant inter-user findings indicate no detected difference rather than demonstrated equivalence. The human cadaver study, though more clinically relevant, represents only time-zero biomechanical performance. Long-term healing outcomes and the biological response to fiber interlocking were tested in ovine RC models but require future evaluation in ongoing clinical studies. The ISP tendon was used instead of the supraspinatus tendon, as it is typically thinner and mechanically weaker, thereby representing a conservative and challenging test environment for augmentation.^18^ However, this substitution may limit the generalizability of the findings, as the supraspinatus, which is most commonly involved in rotator cuff repairs, may respond differently in terms of fixation behavior and failure patterns. The study lacked a conventional patch-augmentation comparator, so superiority over conventional patch fixation cannot be claimed in human tissue. Finally, the limited sample size (n = 5 per group) limits the statistical power for detecting smaller differences between techniques and makes negative findings difficult to interpret.

## Conclusion

In a small human cadaveric model, augmentation with direct patch interlocking substantially improved the mechanical integrity of the suture–tendon construct, even in human tissue. In a preliminary reproducibility assessment, biomechanical results among first-time and experienced users did not differ significantly. These findings are hypothesis-generating and support further, adequate biomechanical and clinical evaluation.

## Disclaimers:

Funding: Cadaveric tendons as well as materials used for the study were funded by ZuriMED Technologies AG.

Conflicts of interest: Ronja Senn, Andrea Möhl, and Cherilyn Camichel are employed by ZuriMED Technologies. Bettina Hochreiter, Ronja Senn, Andrea Möhl, Cherilyn Camichel, and Karl Wieser are shareholders in ZuriMED Technologies. Karl Wieser and Bettina Hochreiter are consultants for ZuriMED. Any additional authors, their immediate families, and any research foundations with which they are affiliated have not received any financial payments or other benefits from any commercial entity related to the subject of this article.
